# Enhanced Temperature Control Method Using ANFIS with FPGA

**DOI:** 10.1155/2014/239261

**Published:** 2014-03-04

**Authors:** Chiung-Wei Huang, Shing-Tai Pan, Jun-Tin Zhou, Cheng-Yuan Chang

**Affiliations:** ^1^Department of Electronic Engineering, Chien Hsin University of Science and Technology, Jhongli 320, Taiwan; ^2^Department of Computer Science and Information Engineering, National University of Kaohsiung, Kaohsiung 811, Taiwan; ^3^Department of Electrical Engineering, Chung Yuan Christian University, Jhongli 320, Taiwan

## Abstract

Temperature control in etching process is important for semiconductor manufacturing technology. However, pressure variations in vacuum chamber results in a change in temperature, worsening the accuracy of the temperature of the wafer and the speed and quality of the etching process. This work develops an adaptive network-based fuzzy inference system (ANFIS) using a field-programmable gate array (FPGA) to improve the effectiveness. The proposed method adjusts every membership function to keep the temperature in the chamber stable. The improvement of the proposed algorithm is confirmed using a medium vacuum (MV) inductively-coupled plasma- (ICP-) type etcher.

## 1. Introduction

Etching process uses a strong acid to cut into the unprotected parts of a metal surface to create a design in intaglio in the metal. It is critical to integrated circuit (IC) design and the semiconductor industry. The etching rate and etching uniformity are directly related to the stability of the controlled temperature of the wafer in the vacuum chamber. As the air pressure in the chamber falls, the temperature of the wafer drops accordingly, degrading the etching process. In the conventional method, a PID controller is utilized to adjust the power of the heater plate to keep the temperature of wafer in the vacuum chamber stable. This method depends on an experienced operator to tune the gains of the PID controller of the heater. The gains must be adjusted when the temperature is varied.

In recent years, researchers have discussed the etching process and analyzed the characteristics of the ICP-type etcher [1–6]. Temperature diagnostics for bell-jar-top ICP has been carried out using atomic emission spectroscopy [2]. The electron temperature depends primarily on pressure [3]. Jang and Lee studied the dependence of inductively coupled plasma systems on pressure and input power and the independence of the electron temperature from the input power [4]. However, these investigations have only verified that the temperature in the chamber is related to the pressure; they do not provide a means of keeping the temperature stable under variations in air pressure. In addition, estimation, measurement, and control of temperature are an important issue for industry applications.

There are also many researches that focus on this topic to improve the efficiency, accuracy, or productivity in terms of monitoring or estimating the temperature within a specific range. In [7], temperature control problems in semiconductor single-crystal rod growth from melt by edge-defined film-fed growth (EFG) technique were investigated. Besides, Li and Meng used temperature decoupling control method based on adaptive PID neural network for a double-level air flow field dynamic vacuum system in [8]. The performance was also good to save the computing load when compared with elaborating a mathematical model for precise control. Girault and Videcoq proposed a multi-input multi-output (MIMO) thermal control problem in real time [9]. The objective is to control temperature at 3 chosen locations. Besides, a cascade control methodology for superheated processes is developed in terms of neural network based PID controller [10]. Also, a feedback control is combined with feedforward control in this design. Gupta et al. presented an active structural vibration control such that the system is robust to temperature variations [11]. In [12], Zheng et al. proposed a method to monitor the strip's transient temperature and accurately controlled the coiling temperature in a hot-rolled strip laminar cooling (HSLC) process. A predictor was also employed to predict the future temperature sequence at the inlet of fine cooling zone to improve the performance of the process.

Numerous researchers have developed applications of ANFIS systems [13–16]. The concept of the ANFIS algorithm was first proposed by Jang in 1993 [13]. This work develops an ANFIS algorithm for tuning the pulse-width modulation (PWM) duty cycle for a heater plate to control the temperature of a chamber under varying pressure. The input signals of the ANFIS algorithm are the present temperature, (*k*), and the variations of both temperature Δ*T*(*k*) and pressure Δ*P*(*k*). A multilayered neural fuzzy system with an adaptive algorithm is utilized in the developed ANFIS system to ensure temperature stability and to minimize the error between the set and present temperatures. A performance surfaceis used to adapt the ANFIS by backpropagation algorithm. The proposed method is realized using an FPGA to generate a PWM signal to control temperature in the vacuum chamber and to improve the etching rate.

This work is organized as follows.  Section 2 reviews the concepts of the proposed algorithm. The structure of an ICP etcher and the methods for designing the ANFIS algorithm are also given.  Section 3 presents various experimental results and discusses the performance of the proposed method and a commercial product of temperature controller. The introduction to the experimental setup, including the FPGA, the temperature and pressure measurement tools are also made. Finally, Section 4 draws some conclusions.

## 2. Control Method

Keeping the temperature in the vacuum chamber stable is one of the most important means of ensuring favorable etching rate. However, when the pressure in the chamber changes, the temperature also changes. Therefore, an ANFIS algorithm is designed to stabilize the temperature of the heater plate in the chamber to achieve a stable temperature.

### 2.1. ICP-Type Etcher

**Figure 1:**
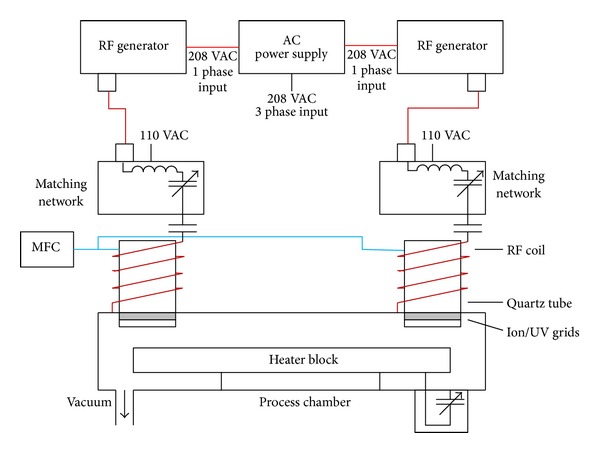
ICP-type etcher.

Etching machines can be divided into reactive ion etchers (RIE) and high-density plasma (HDP) etchers based on their operating method. However, HDP etchers are currentlyfavored in the semiconductor industry. High-density plasma can be generated as ICP, electron cyclotron resonance plasma (ECR), and helicon wave plasma. This work develops an algorithm for controlling an ICP etcher.  Figure 1 displays the basic structure of an ICP etcher. The main components of this etcher include the RF generator, matching network, RF coil, quartz tube, mass-flow controller (MFC), vacuum system, and heater block. The wafer is placed on the heater block for etching.

In an ICP etcher, the control of the temperature in the vacuum chamber influences the etching rate. The conventional method involves using a thermocouple to measure the temperature of the heater block and the PID method to control the heater. Since the chamber belongs to medium vacuum (MV) stage, the temperature in the air responds very rapidly to any variation of pressure. The variation of temperature can be determined from the change of pressure using the Poisson equation,

(1) $$\begin{array}{c} \frac{T_{1}}{T_{2}}={\left(\frac{P_{1}}{P_{2}}\right)}^{\left(\gamma -1\right)/\gamma }, \end{array}$$

where *γ* ≈ 1.4 is the specific heat capacity and *T*_1_ and *T*_2_ denote the temperatures at pressures *P*_1_ and *P*_2_ in the vacuum chamber, respectively [14]. All materials inside the chamber are metal, which transfer heat very rapidly. Therefore, achieving a stable temperature using the PID method as the pressure varies is difficult. The variation in pressure also resulted in the change in the temperature of the wafer, degrading the etching rate and the performance.

### 2.2. ANFIS Algorithm

By combining the fuzzy inference system (FIS) and the neural network, this algorithm can tune the membership function of FIS automatically and be implemented model-free during controller design. The temperature error *e*(*k*), variation of temperature error Δ*e*(*k*), and air pressure variation Δ*P*(*k*) at time *k* are the input signals of the proposed neural fuzzy network, where *e*(*k*) = *T*(*k*) − *T*_*s*_, Δ*e*(*k*) = *e*(*k*) − *e*(*k* − 1), and *T*_*s*_ is the set temperature of the etcher. A PWM duty cycle signal to drive the heater plate in the chamber is thus derived. The performance index is

(2) $$\begin{array}{c} \phi \left(k\right)=e^{2}\left(k\right), \end{array}$$

where the gradient estimation method [15] is utilized to tune the free variables of the proposed algorithm.

**Figure 2:**
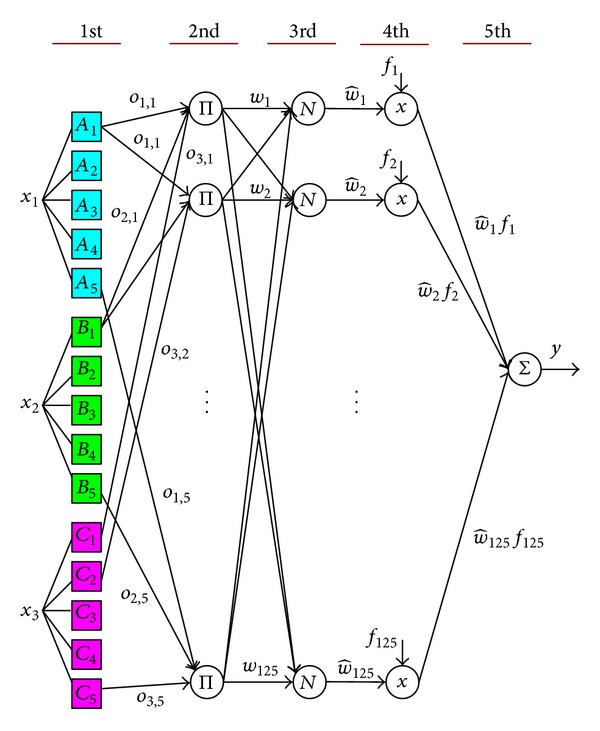
ANFIS algorithm.

Figure 2 shows the proposed neural fuzzy network. Let $\left[\begin{array}{ccc} x_{1} & x_{2} & x_{3} \end{array}\right]=\left[\begin{array}{ccc} e(k) & \Delta e(k) & \Delta P(k) \end{array}\right]$ be the input signals. Two hidden layers perform the operations of the fuzzifier and the fuzzy inference engine. An output node completes the defuzzifier. The connections between consecutive layers are weighted, and the weighting values will be updated based on the steepest descent method.

**Figure 3:**
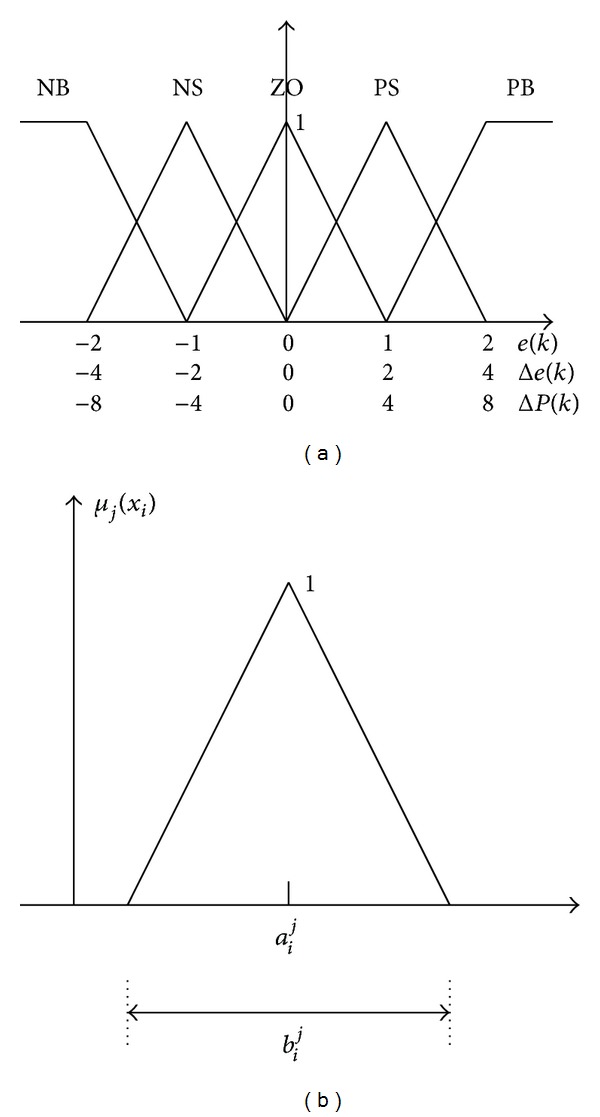
Membership functions. (a) Linguistic terms of each input variable. (b) Parameter settings for triangular membership function.

In the first layer, three input variables $\left[\begin{array}{ccc} x_{1} & x_{2} & x_{3} \end{array}\right]=\left[\begin{array}{ccc} e(k) & \Delta e(k) & \Delta P(k) \end{array}\right]$, each with five linguistic terms, are connected as presented in Figure 2. Therefore, this layer has 15 nodes to fuzzify the input variables. Let the linguistic terms associated with all variables be represented as triangular sets, as presented in Figure 3:

(3) $$\begin{array}{c} \mu_{j}\left(x_{i}\right)=1-2\frac{\left|x_{i}-a_{i}^{j}\right|}{b_{i}^{j}}\equiv o_{i,j},\;\left(i=1,2,3,\;\;j=1\sim 5\right), \end{array}$$

where *a*_*i*_ and *b*_*j*_ are the parameters of each triangle and the true values *μ*_*j*_(*x*_*i*_) ≡ *o*_*i*,*j*_ denote fuzziness. Accordingly, 5∗5∗5 = 125 fuzzy rules are obtained. For example,

(4) Rl  :  If  x1  is  Ai,l  AND  x2  is  Bj,l  AND  x3  is  Ck,l, then  y  is  fl  (i,j,k=1,…,5;l=1,…,125),

where *A*_*i*,*l*_, *B*_*j*,*l*_, and *C*_*k*,*l*_ are the linguistic states associated with respective input variables and *f*_*l*_ is the linguistic state associated with the duty cycle of the PWM signal forthe heater. The second layer computes the minimum of each true value of the antecedent parts to execute the AND operation in (4). Consider the first fuzzy rule *R* ^1^ as an example: the fuzziness of the antecedent part is *w*_1_ = *o*_1,1_ · *o*_2,1_ · *o*_3,1_. Hence, the third layer calculates the normalized fuzziness for each rule by

(5) $$\begin{array}{c} {\hat{w}}_{l}=\frac{w_{l}}{w_{1}+w_{2}+\cdots +w_{125}},\;\left(l=1,\ldots ,125\right). \end{array}$$

**Figure 4:**
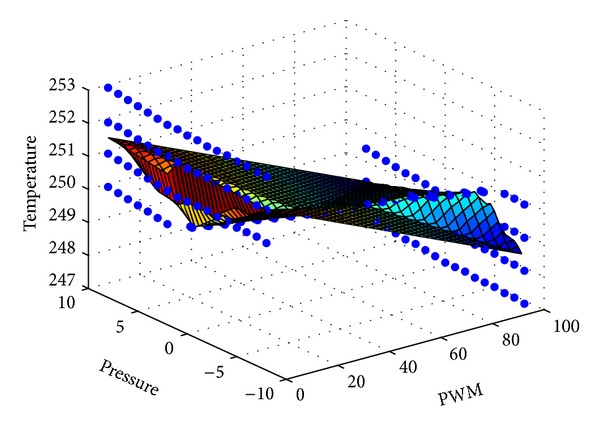
Performance surface for etching process in chamber.

The last two layers generate the output signal of the neural fuzzy system,

(6) $$\begin{array}{c} y=\sum_{l=1}^{125}{\hat{w}}_{l}\cdot f_{l} \end{array}$$

which is the PWM duty cycle to control the heater plate in the chamber.  Figure 4 plots the best performance surface of the etching process for various air pressures and temperatures in the chamber. Pressure variation importantly affects the etching process. Therefore, the algorithm for tuning the PWM signal involves pressure variation. The goal herein is to ensure that the operating points are close to the flat area of the performance surface. Therefore, the circles in the figure, which were specified by experienced engineers who know how to perform the PWM for the heater in the chamber, are chosen to specify the fuzzy singleton values *f*_1_ ~ *f*_125_.

In the developed design, the fuzzy membership functions and the weights of the neural fuzzy networks are adjustable. The parameters of the triangular membership functions are

(7) aij(k+1)=aij(k)−η∂E∂aij,bij(k+1)=bij(k)−η∂E∂bij,

based on the steepest descent method, where

(8) ∂E∂aij=∂E∂T(k)·∂T(k)∂y·∂y∂wl·∂wl∂μj(xi)·∂μj(xi)∂aij=2[T(k)−Ts]·F(ΔP,y) ·∑l=1125(flwl)·wioi,j·sgn⁡(xi−aij)2bij,∂E∂bij=∂E∂T(k)·∂T(k)∂y·∂y∂wl·∂wl∂μj(xi)·∂μj(xi)∂bij=2[T(k)−Ts]·F(ΔP,y) ·∑l=1125(flwl)·wioi,j·2|xi−aij|(bij)2.

**Figure 5:**
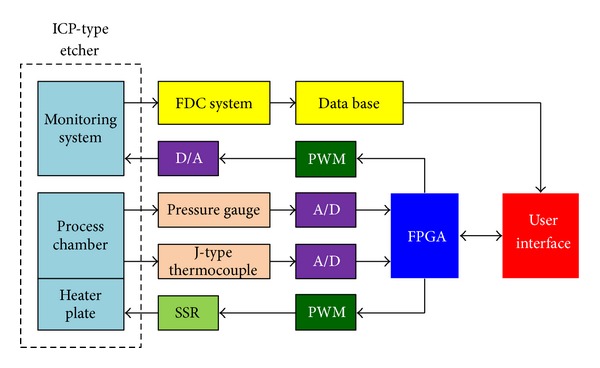
Block diagram of etcher system.

The performance surface in Figure 5 is utilized to compute ∂*T*(*k*)/∂*y* ≡ *F*(Δ*P*, *y*) in (8). The surface fitting tool in Matlab is used to compute ∂*T*(*k*)/∂*y* by fitting *T*(*k*) as a third-order polynomial based on the root mean square error (RMSE) method. Hence,

(9) T(k)≡f(ΔP,y)=c0+c1ΔP+c2y+c3ΔP2+c4ΔP·y +c5y2+c6ΔP2·y+c7ΔP·y2+c8y3+c9ΔP3,

where *c*_0_ ~ *c*_9_ are the respective weights of every item; therefore,

(10) F(ΔP,y)≡∂T(k)∂y=∂f(ΔP,y)∂y=c2+c4ΔP+2c5y+c6ΔP2+2c7ΔP·y+3c8y2.

Equation (10) can be substituted into (8) to realize the proposed ANFIS algorithm.

## 3. Experimental Results 

**Figure 6:**
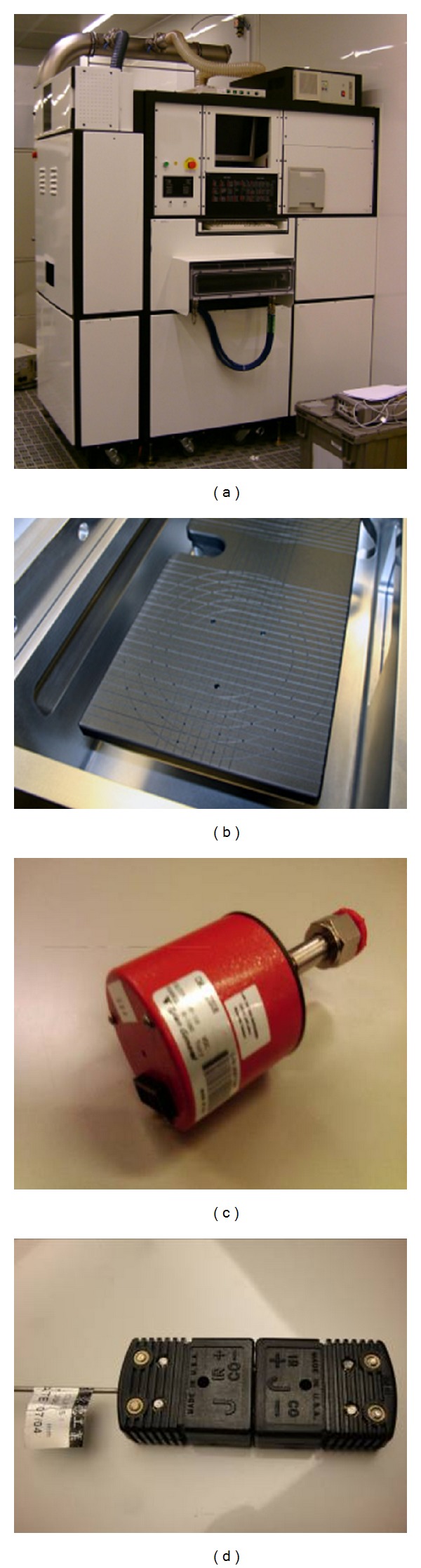
Components of experimental setups. (a) ICP etcher, (b) heater plate, (c) pressure gauge, and (d) J-type thermocouple.

To illustrate the performance of the proposed ANFIS system, the following conditions are considered. Figure 5 shows the block diagram of the overall system, where an FPGA is the controller and the ICP etchers (MATTSON Aspen II) are embedded with a pressure gauge (made by Tylan General Co. Ltd., model CMLB-21S06, for input 0~100 torr and output 0~10 vdc) and a J-type thermocouple to measure the pressure in the process chamber and the temperature on the heater plate. Figures 6(a)–6(d) show the ICP etcher, heater plate, pressure gauge, and J-type thermocouple, respectively. The FPGA herein is the Spartan 3E Starter Kit by Xilinx, which is programmed using the Verilog language to develop the proposed algorithm. The parameters of *F*(Δ*P*, *y*) are *c*_2_ = −0.1307, *c*_4_ = −3.843∗10^−2^, *c*_5_ = −1.271 × 10^−3^, *c*_6_ = 6.251 × 10^−2^, *c*_7_ = 3.585 × 10^−4^, and *c*_8_ = −3.46 × 10^−6^ which were obtained by Matlab.

**Figure 7:**
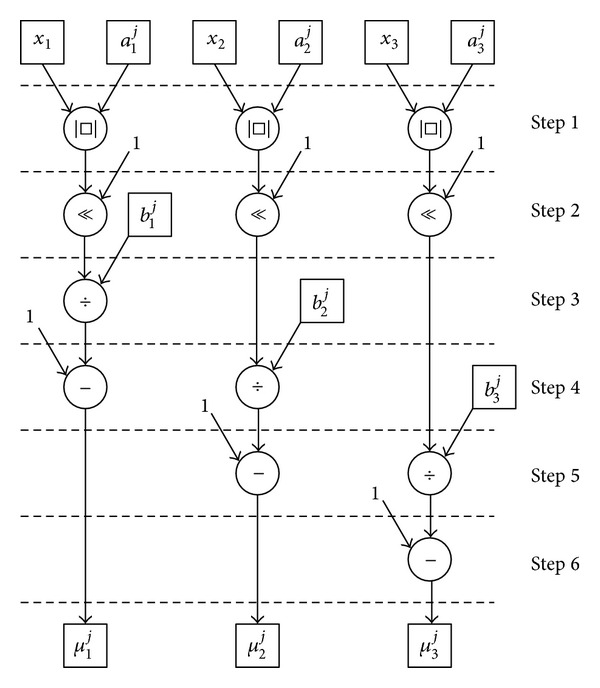
Computing method of FPGA.

The multiplication and division operations impose a large computational load in FPGA. To reduce this load, the algorithm performs only two multipliers and one divider used. Using (3) yields the operation of FPGA, as presented in Figure 7. Step 1 computes the absolute value of *x*_*i*_ − *a*_*i*_^*j*^ and step 2 shifts the data by one bit to multiply two. Since only one divider is conducted, the divisions of *b*_*i*_^*j*^ are performed separately for |*x*_*i*_ − *a*_*i*_^*j*^|  (*i* = 1,2, 3) in steps 3–5, and then one is subtracted from the result in the following step. Some terms, such as the denominator in (5), are used several times in the algorithm and the results thus obtained can be saved at registersto accelerate signal processing. Therefore, control task can be performed very rapidly using the FPGA.

The PWM duty cycle time was initially 1 second. When the output of FPGA is 90, the duty cycle changes to 1∗90% = 0.9 s. In this work, the clock rate of FPGA (500 MHz = 5 × 10^7^ Hz) as a counter is set and the first output is one. When the FPGA has counted to 0.9∗5 × 10^7^ = 4.5 × 10^7^, the system output remainszero until the FPGA counts to 5 × 10^7^ one cycle. Therefore, the PWM signal can be easily produced.

Several experiments are carried out to confirm the performance of the proposed scheme. A leading commercial product (WATLOW series 988) is used to compare the performance with the proposed approach for controlling the temperature of the chamber. The learning rate, used in (7), is 0.7. The initial values of *a*_*i*_^*j*^ and *b*_*i*_^*j*^ are those used in Figure 3. The temperature is measured every 2 seconds and the results obtained using the proposed ANFIS method are compared with those obtained using the commercial product.

**Figure 8:**
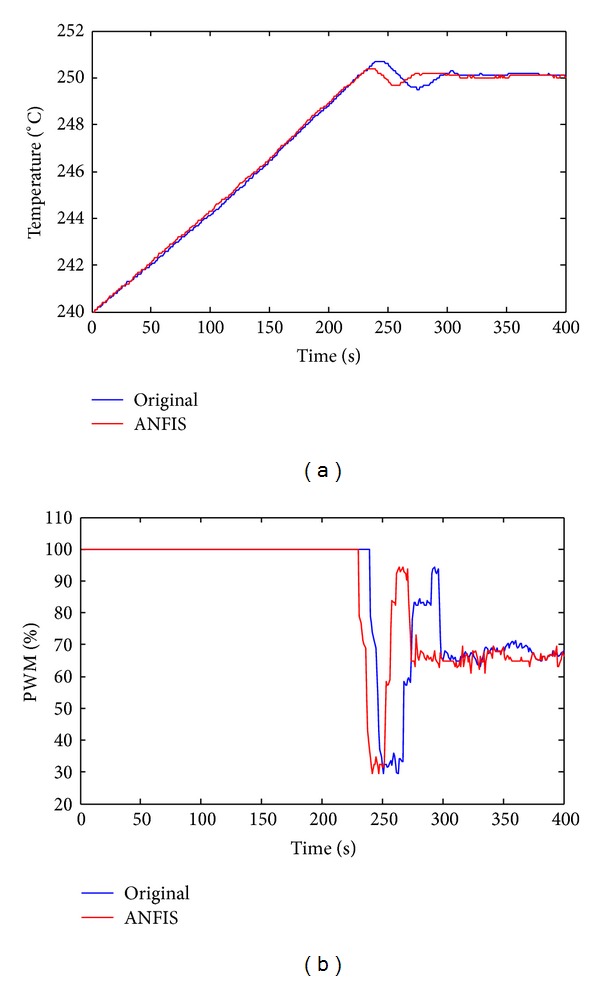
Experiments of increasing temperature without variation of air pressure. (a) Temperature. (b) PWM (red: ANFIS; blue: original product).

In the first experiment, the initial temperature in the process chamber is 240°C, but the temperature is set to 250°C (*T*_*s*_ = 250) and the air pressure is held constant (Δ*P* = 0).  Figure 8 presents the results that are thus obtained. In Figure 8(a), both the original and the proposed method use a 100% PWM duty cycle for the heater plate; therefore, both methods yield almost equal heating rates. However, the proposed ANFIS method provides faster convergence than the commercial product, as presented in Figure 8(a).  Figure 8(b) plots the results of the PWM duty cycle. It proves that the proposed method converges faster than the use of the original product.

**Figure 9:**
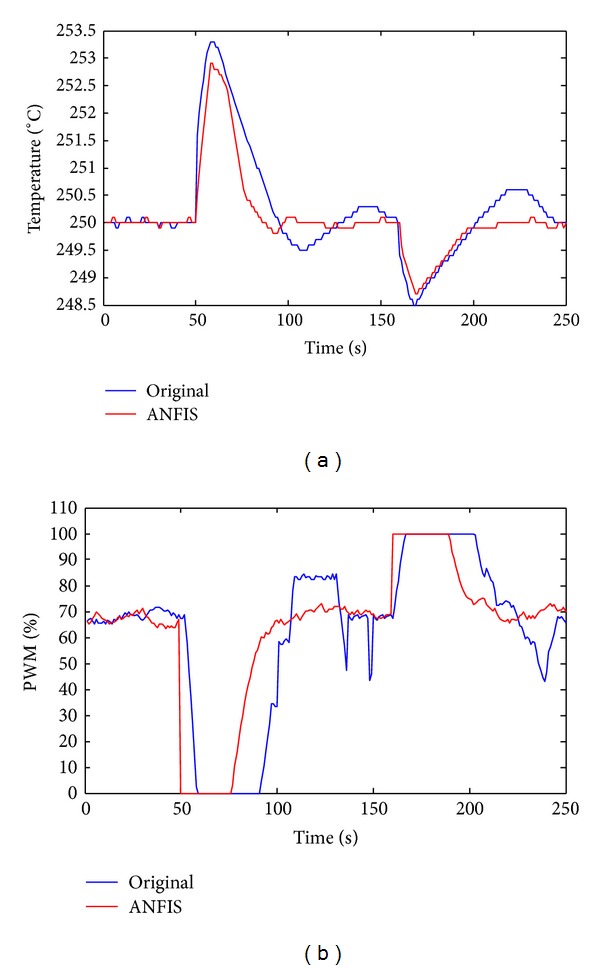
Experiments with the variation of air pressure. (a) Temperature. (b) PWM (red: ANFIS; blue: original product).

The following experiment examines the performance at 250°C with the pressure in the process chamber increased by 10 torr at 50 s and decreased by 10 torr at 160 s. The results obtained using the proposed ANFIS are better than those obtained using the commercial product. Also, increasing the air pressure increased temperature, confirming the Poisson equation.  Figure 9(a) plots the results of temperature control and Figure 9(b) shows the results of the PWM cycle. Since there is no cooler system in etcher, both the proposed ANFIS and commercial products turn off the heater (PWM = 0) when the pressure in the chamber increases at 50 s. However, the proposed method that involves FPGA responds very quickly, with a PWM of almost zero at the same time. When the pressure decreases to 10 torr at 160 s, the proposed method turns on the heater faster than does the commercial product and convergence is very rapid.

The last experiment tests the practical case in etching process. In the semiconductor industry, the pressure in the chamber may change frequently because the operator must sometimes open the process chamber, reducing the pressure. Other operations may also increase the pressure in the chamber. Therefore, in this experiment, the pressure in the chamber is set to decrease by 5 torr at 20 s; to increase by 5 torr at 40 s, to increase by 3 torr at 100 s and to decrease by 6 torr at 130 s.

**Figure 10:**
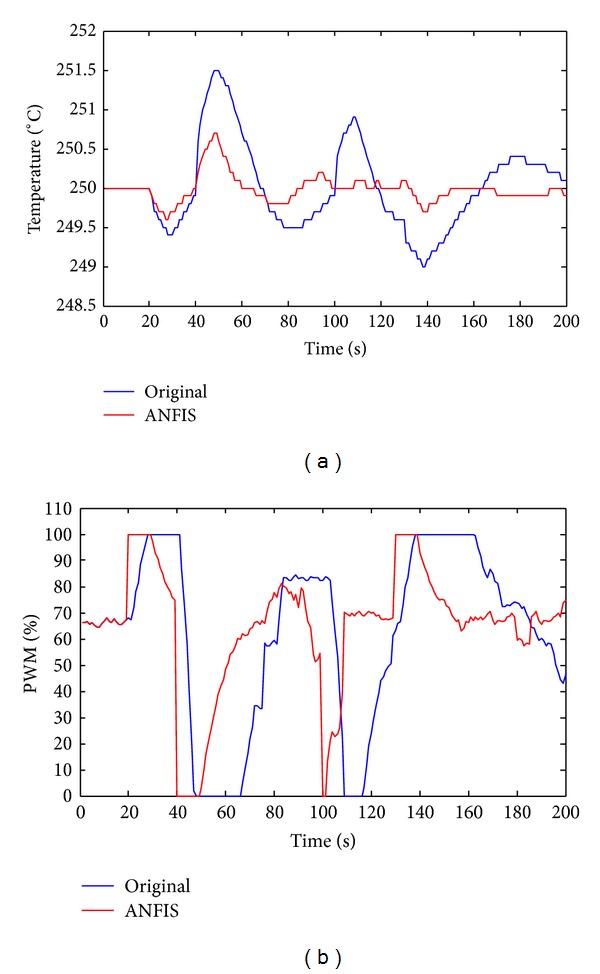
Experiments with frequently variation of pressure. (a) Temperature. (b) PWM (red: ANFIS; blue: original product).

Figure 10 plots the results of temperature control. Clearly, the commercial product cannot maintain a stable temperature during the process. When the pressure varies at 20, 40, 100, and 130 s, the original controller controls poorly, as presented in Figure 10(a) in blue. However, the proposed method, which uses FPGA, controls the temperature effectively.  Figure 10(b) plots the results of the PWM duty cycle for the heater plate. These results demonstrate the rapid response of the proposed ANFIS method with FPGA.

## 4. Conclusions

This work presents the ANFIS algorithm for controlling the temperature in the vacuum chamber in an etching machine. The reduction of the computing load and the realization of the proposed algorithm by FPGA are described. The proposed method very rapidly responds to variations in pressure in the vacuum chamber very and converges quickly to a set temperature, ensuring a favorable etching rate and etching performance. Practical applications are studied to confirm the improvement that is provided by the proposed method.
